# Biogenic Silver Nanoparticles Synthesized by *Lysinibacillus xylanilyticus* MAHUQ-40 to Control Antibiotic-Resistant Human Pathogens *Vibrio parahaemolyticus* and *Salmonella Typhimurium*

**DOI:** 10.3389/fbioe.2020.597502

**Published:** 2020-12-16

**Authors:** Md. Amdadul Huq

**Affiliations:** Department of Food and Nutrition, College of Biotechnology and Natural Resource, Chung-Ang University, Anseong, South Korea

**Keywords:** eco-friendly synthesis, AgNPs, *Lysinibacillus xylanilyticus* MAHUQ-40, antimicrobial activity, human pathogens

## Abstract

The present study highlights a simple and eco-friendly method for the biosynthesis of silver nanoparticles (AgNPs) using *Lysinibacillus xylanilyticus* strain MAHUQ-40. Also, the synthesized AgNPs were used to investigate their antibacterial activity and mechanisms against antibiotic-resistant pathogens. Biosynthesis of AgNPs was confirmed by ultraviolet–visible spectroscopy, and then, they were characterized by field emission-transmission electron microscopy (FE-TEM), X-ray diffraction (XRD), dynamic light scattering (DLS), and fourier transform-infrared (FTIR). The toxicity of AgNPs against two pathogenic bacteria was evaluated. The UV–vis spectral scanning showed the peak for synthesized AgNPs at 438 nm. Under FE-TEM, the synthesized AgNPs were spherical with diameter ranges from 8 to 30 nm. The XRD analysis revealed the crystallinity of synthesized AgNPs. FTIR data showed various biomolecules including proteins and polysaccharides that may be involved in the synthesis and stabilization of AgNPs. The resultant AgNPs showed significant antibacterial activity against tested pathogens. The MICs (minimum inhibitory concentrations) and MBCs (minimum bactericidal concentrations) of the AgNPs synthesized by strain MAHUQ-40 were 3.12 and 12.5 μg/ml, respectively, against *Vibrio parahaemolyticus* and 6.25 and 25 μg/ml, respectively, against *Salmonella Typhimurium*. FE-TEM analysis showed that the biogenic AgNPs generated structural and morphological changes and damaged the membrane integrity of pathogenic bacteria. Our findings showed the potentiality of *L. xylanilyticus* MAHUQ-40 to synthesis AgNPs that acted as potent antibacterial material against pathogenic bacterial strains.

## Introduction

Nanotechnology is developing rapidly because of its utilization in different fields, especially for the production of beneficial nanoproducts (Sarker and Nahar, 2017). Metal nanoparticles (NPs) show unique magnetic and optical properties and have wonderful mechanical strength and large surface area with low melting points, which allows for use of metal NPs in different fields of science including medicine and industry (Horikoshi and Serpone, 2013). Also, these characteristics of NPs make them more valuable in diagnostics, bio-imaging, and drug delivery systems (Baetke et al., 2015; Huerta-Aguilar et al., 2019). Among metal NPs, silver NPs (AgNPs) have gained remarkable attraction because of their unique biological and physicochemical characteristics (Chaloupka et al., 2010).

There are various methods for the synthesis of NPs including chemical and physical methods, but the generation of numerous hazardous substances has limited their use (Gour and Jain, 2019). Because of the increased use of NPs in various fields of science, researchers are trying to develop green techniques for the biosynthesis of NPs and to improve the biocompatibility of NPs (Princy and Gopinath, 2018; Tamarov et al., 2018). Biological synthesis has several advantages including high stability, and less toxicity against healthy cells and free from toxic by-products synthesis (Thakkar et al., 2010; Raja et al., 2017). Moreover, biosynthesis of NPs is a low-cost approach (Noruzi et al., 2011). Furthermore, comparing with physicochemical approaches, green techniques are able to synthesize NPs with different shapes and unique features under controlled conditions like pH, temperature, etc., (Thakkar et al., 2010; Noruzi et al., 2011). Several techniques have been raised for the eco-friendly synthesis of different NPs using plants (Aygün et al., 2019a), bacteria (Akter and Huq, 2020), fungi (Aygün et al., 2019b), and algae (De-Aragao et al., 2016). Use of microorganisms for the green synthesis of NPs is a cost effective, facile, and eco-friendly process. Bacteria have been gaining more interest for researchers for the biosynthesis of metal NPs because of their easy handling and manipulation. Moreover, bacterial cells contain numerous bioactive compounds including proteins, pigments, and polysaccharides that can be used as reducing and capping agents during synthesis process (Cepoi et al., 2014; Shukla and Iravani, 2017). Several bacteria have been reported to be able to synthesize silver NPs including *Pseudomonas aeruginosa* (Shivakrishna et al., 2013), *Microvirga rosea* (Huq, 2018), *Bacillus cereus* (Prakash et al., 2011), and *Bacillus methylotrophicus* (Wang et al., 2016).

Emergence of multidrug-resistant (MDR) bacteria is a serious concern for public health (Holmes et al., 2016). To control these MDR bacteria, it is essential to develop alternative antibacterial agents (Jain et al., 2009). Biosynthesized AgNPs could be the alternative antibacterial agents to control the MDR bacteria, as AgNPs exhibit strong bactericidal activity (Song and Kim, 2009; Abdel-Raouf et al., 2019). AgNPs have potent inhibitory activity against pathogenic bacteria because of their very small sizes with big surface area, which accelerate the wide interactions between AgNPs and pathogens (Abdel-Raouf et al., 2017; Huq, 2020). In this study, *Lysinibacillus xylanilyticus* MAHUQ-40 was isolated from soil sample of a persimmon garden, and used for the eco-friendly synthesis of AgNPs. The resultant AgNPs were characterized and their antimicrobial activity in the mechanism level was investigated against two MDR pathogenic bacteria *Vibrio parahaemolyticus* and *Salmonella Typhimurium*.

## Materials and Methods

### Materials

All culture media were bought from Difco, MB Cell (Seoul, South Korea). AgNO_3_ (Silver nitrate) was bought from Sigma-Aldrich. Two pathogens *V. parahaemolyticus* (ATCC 17802) and *S. Typhimurium* (ATCC 14028) were collected from the American Type Culture Collection (ATCC). Standard antibiotics discs: Oleandomycin (OL15) 15 μg/disc, erythromycin (E15) 15 μg/disc, lincomycin (MY15) 15 μg/disc, vancomycin (VA30) 30 μg/disc, and penicillin G (P10) 10 μg/disc were gained from Oxoid Ltd., England.

### Isolation and Characterization of Bacteria

The soil sample from a persimmon garden was collected in a 50-ml conical tube from Anseong, South Korea. The sample was serially diluted using NaCl solution (0.8%), spread onto Reasoner’s 2A (R2A) agar, and then incubated for 48 h at 30°C to obtain colonies. The AgNP synthesis ability was screened by culturing the individual colonies on R2A agar plate supplemented with 1 mM filter-sterilized AgNO_3_. The positive colonies were sub-cultured and collected in the pure form. Molecular identification of the positive strain was done through 16S rRNA gene sequencing. Extraction of genomic DNA, amplification of 16S rRNA gene from genomic DNA, and sequencing of this amplified gene were done according to the previous description (Huq, 2020). The 16S rRNA gene sequence (1,479 bp) was assembled by SeqMan software and the 16S rRNA gene sequences of close type strains were collected from the EzTaxon-eserver (Kim et al., 2012). Phylogenetic tree was created using MEGA6 program (Tamura et al., 2013) and neighbor joining algorithm (Saitou and Nei, 1987) to detect the phylogenetic position of strain MAHUQ-40.

### Eco-Friendly Synthesis of AgNPs

For the eco-friendly synthesis of AgNPs, the isolated bacterial strain MAHUQ-40 was added into 100 ml of R2A broth and incubated in a shaking incubator with 160 rpm at 30°C for 48 h. Then, the culture supernatant was collected through centrifugation at 9,000 rpm for 15 min. The culture supernatant was mixed with 100 μl of AgNO_3_ solution (1 M), with a final concentration of 1 mM of AgNO_3_ and then the mixture was again incubated in a shaking incubator for 48 h (160 rpm, 30°C). The AgNP synthesis was observed through visual inspection according to the previous description (Huq, 2020). After the incubation period, the reaction mixture was centrifuged with high speed (14,000 rpm) for 20 min to collect the synthesized AgNPs. The synthesized AgNPs were washed thoroughly with distilled water to remove the undesired components, and finally, the eco-friendly synthesized AgNPs were obtained in the pellet form.

### Characterization of Synthesized AgNPs

During the biological synthesis of AgNPs, 200 μl of reaction mixtures was withdrawn and monitored using UV-VIS (Ultraviolet–visible) spectroscopy (Optizen POP, Mecasys) with a wavelength range from 300 to 800 nm. FE-TEM (field emission–transmission electron microscopy) was used to analyze the shape and size of biosynthesized AgNPs. Elemental compositions and purity of green synthesized AgNPs were examined using EDX (energy dispersive X-ray) spectroscopy and SAED (selected area diffraction) pattern operated by TEM-2100F (JEOL). A drop of AgNPs suspension was placed on the carbon-coated copper grid and then, dried under infrared lamp before analysis. The crystallinity of the biosynthesized AgNPs powder was investigated by X-ray diffraction (XRD) analysis (D8 Advance, Bruker, Germany) over the range of 30–80° (2θ), operated using CuKα radiation, at 40 kV and 40 mA with a scan speed 6°/min.

The functional groups of synthesized AgNPs were identified by FTIR (fourier transform-infrared). FT-IR was performed using air-dried AgNPs powders and Perkin Elmer Fourier transform infrared spectrometer. Spectra of FTIR were collected across the range of 400–4,000 cm^–1^ with a resolution of 4 cm^–1^. The size distribution profile of the synthesized NPs was studied using dynamic light scattering (DLS) with Malvern Zetasizer Nano ZS90 (Malvern Instruments, Worcestershire, United Kingdom). The polydispersity index (PDI) and hydrodynamic diameters were calculated at a 12° angle and at 25°C. A dispersive medium of pure water was used as reference with a viscosity of 0.8878, a refractive index of 1.3328, and a dielectric constant of 78.3 (Akter and Huq, 2020).

### Antimicrobial Activity

The antimicrobial activity of the synthesized AgNPs was carried out using the disc diffusion method (Singh et al., 2018) against pathogenic microorganisms *V. parahaemolyticus* and *S. Typhimurium* on Nutrient agar (NA) supplemented with 3% NaCl and Mueller–Hinton agar (MHA), respectively. One hundred microliters of overnight cultured each pathogenic strain was spread on the agar plate by spreader. Then, 30 μl of both 500 and 1,000 ppm of synthesized AgNPs solution was added over each disk and incubated for 24 h at 37°C. After the incubation period, the zones of inhibition were calculated around each disc. Similarly, the antibacterial efficacy of some commercial antibiotics including penicillin G (10 μg/disc), oleandomycin (15 μg/disc), erythromycin (15 μg/disc), lincomycin (15 μg/disc), and vancomycin (30 μg/disc) has been tested against *V. parahaemolyticus* and *S. Typhimurium*. The inhibition zones were calculated after 24 h of incubation. This experiment was carried out in triplets.

### Determination of MIC and MBC

The MIC (minimum inhibitory concentration) of eco-friendly synthesized AgNPs was measured using broth micro dilution technique (Du et al., 2019). *V. parahaemolyticus* strain was grown in nutrient broth (NB) supplemented with 3% NaCl and *S. Typhimurium* strain was grown in MH (Mueller–Hinton) broth for overnight at 37°C and the turbidity was fixed around 1 × 10^6^ CFUs/ml. One hundred microliters of test bacterial (1 × 10^6^ CFUs/ml) suspension was added into a 96-well ELISA plate. Then, an equal volume of AgNPs solution with various concentrations (1.56–100 μg/ml) was added, and finally, the plates were incubated in a 37°C incubator for 24 h. Every 3 h of interval, the absorbance (at 600 nm) was taken using ELISA plate reader (LabTech 4000). MBC was determined by streaking of 10 μl of each set on agar plate and again incubated for 24 h at 37°C. The culture plates were watched by the naked eye to determine the minimum bactericidal concentration (MBC) that blocked bacterial growth (Ansari et al., 2018).

### Morphological Evaluation of Treated Cells by FE-SEM

The structural and morphological changes of strain *V. parahaemolyticus* and *S. Typhimurium* were examined by FE-SEM. Cells at logarithmic growth phase (around 1 × 10^7^ CFU/ml) were treated with biosynthesized AgNPs at the concentration of 1 × MBC. As control, both pathogens were treated with 0.85% NaCl solution instead of AgNPs. PBS buffer was used to wash the overnight treated cells. Glutaraldehyde (2.5%) was used for 4 h to fix the cells and then washed several times using PBS. The cells were again fixed by 1% osmium tetroxide and washed again using PBS. Then, the cells were dehydrated at room temperature by various concentrations of ethanol from 30 to 100% for 10 min (Ansari et al., 2018). The dehydrated cells were dried by a desiccator and finally the samples were installed on SEM metallic stubs and coated with gold. The morphological and structural alterations of the cells were seen by FE-SEM (JSM-7100F, JEOL, Japan).

## Results and Discussion

### Identification of AgNPs Producing Bacteria

**FIGURE 1:**
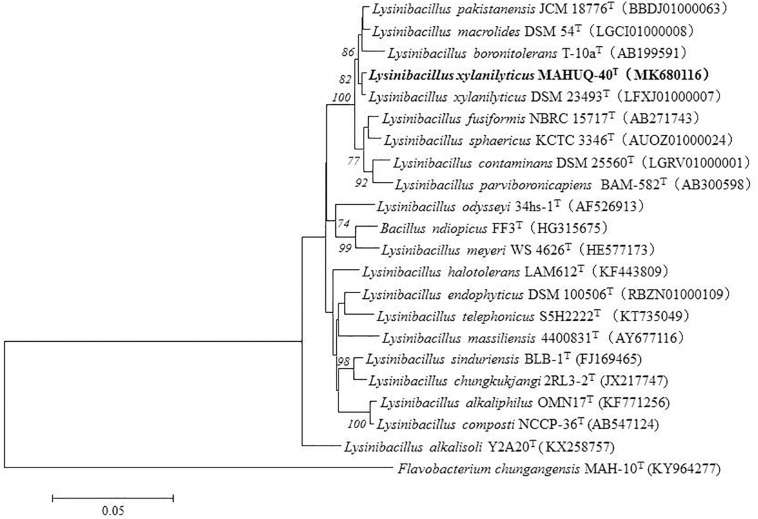
The phylogenetic tree constructed by neighbor-joining (NJ) algorithm on the basis of 16S rRNA gene sequences, showing phylogenetic relationships of strain MAHUQ-40^T^ and members of genus *Lysinibacillus*. Bootstrap values more than 70% based on 1,000 replications are shown at branching points. Scale bar, 0.05 substitutions per nucleotide position.

The size of the 16S rRNA gene sequence of isolated strain MAHUQ-40 was 1,479 bp. This sequence was analyzed and submitted to NCBI (NCBI accession number: MK680116). According to this sequence (16s rRNA), strain MAHUQ-40 showed 99.66% similarity to *L. xylanilyticus*. The phylogenetic tree also revealed that strain MAHUQ-40 was associated with the genus *Lysinibacillus* and created a group with the members of this genus (Figure 1). Strain MAHUQ-40 has been preserved to Korean Agriculture Culture Collection (deposition number: KACC 21239).

### Eco-Friendly and Facile Synthesis of AgNPs

**FIGURE 2:**
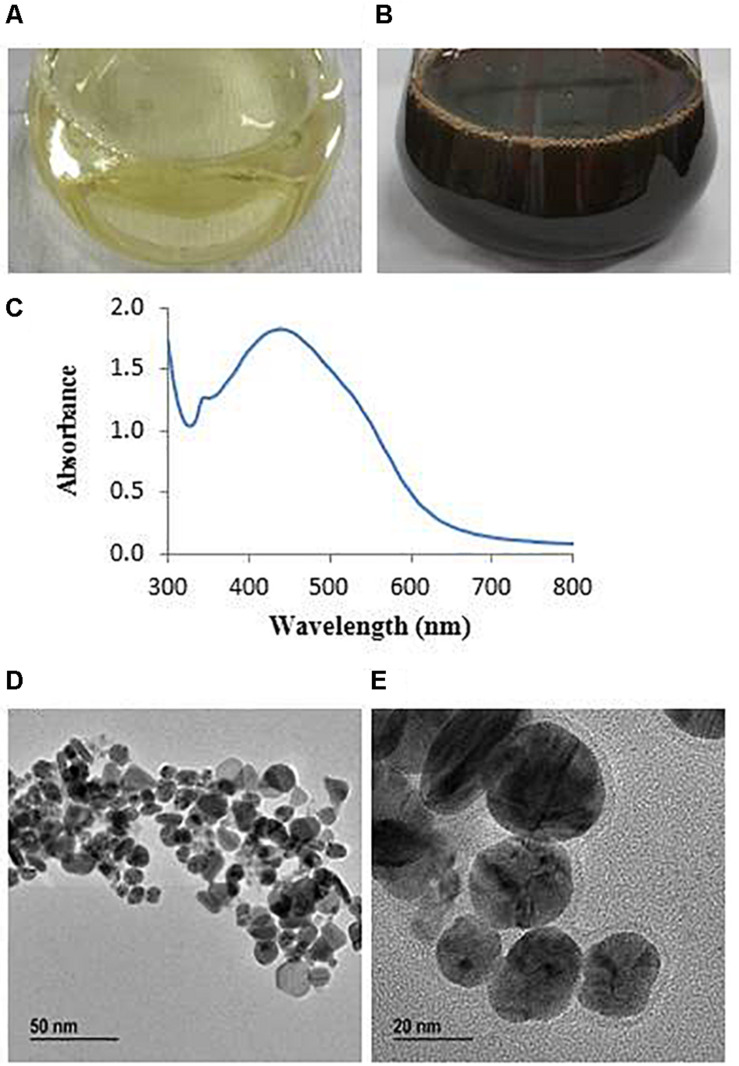
R2A broth with AgNO_3_ as control **(A)**, synthesized AgNPs **(B)**, UV–vis spectra **(C)**, and FE-TEM images of synthesized silver nanoparticles **(D,E)**.

The synthesis of AgNPs from AgNO_3_ was monitored by the change of color from watery yellow to dark brown (Huq, 2020). The color was changed because of the surface plasmon resonance by synthesized AgNPs (Mulvaney, 1996). The color of MAHUQ-40 culture supernatant changed to dark brown within 48 h (Figures 2A,B). The methodology of the present work was extracellular, simple, green, and cost-effective. To date, the accurate mechanism of biosynthesis of NP is weakly understood. According to El-Batal et al. (2013), NADH-dependent reductases play an important role in AgNP biosynthesis. El-Naggar et al. (2016) also reported that cellular molecules like amino acids, enzymes, proteins, pigments, and carbohydrates have been involved in the biosynthesis process of AgNPs. Another report revealed that biosynthesis of NPs involves two steps (Mahdieh et al., 2012). In the first step, the metal salts come in contact with the microbial cells, and in the second step, the metal ions are reduced to metal NPs by secreting reductase enzymes by the microbial cells. The extracellular synthesis method is very easy, cost-effective, and convenient compared to the intracellular method, which requires more difficult and complex steps for purification. In this study, the extracellular method was used for the eco-friendly synthesis of AgNPs using *L. xylanilyticus* MAHUQ-40.

### Characterization of Eco-Friendly Synthesized AgNPs

The AgNPs showed a peak at 438 nm (Figure 2C), which revealed that AgNPs were fruitfully synthesized. Absorbance across the range around 400–500 nm is typical for AgNPs (Huq, 2020). Previous reports showed that AgNPs synthesized from cyanobacterium *Oscillatoria limnetica* and *Oscillatoria willei* have absorption peaks at 426 and 450 nm, respectively (Ali et al., 2011; Hamouda et al., 2019). The lower wavelength value of absorption peak revealed that smaller-sized spherical NPs were produced (Govindaraju et al., 2009), which suggested that *L. xylanilyticus* MAHUQ-40 may synthesize small-sized AgNPs. Our results indicated that *L. xylanilyticus* MAHUQ-40 may be a promising candidate for the biosynthesis of AgNPs.

**FIGURE 3:**
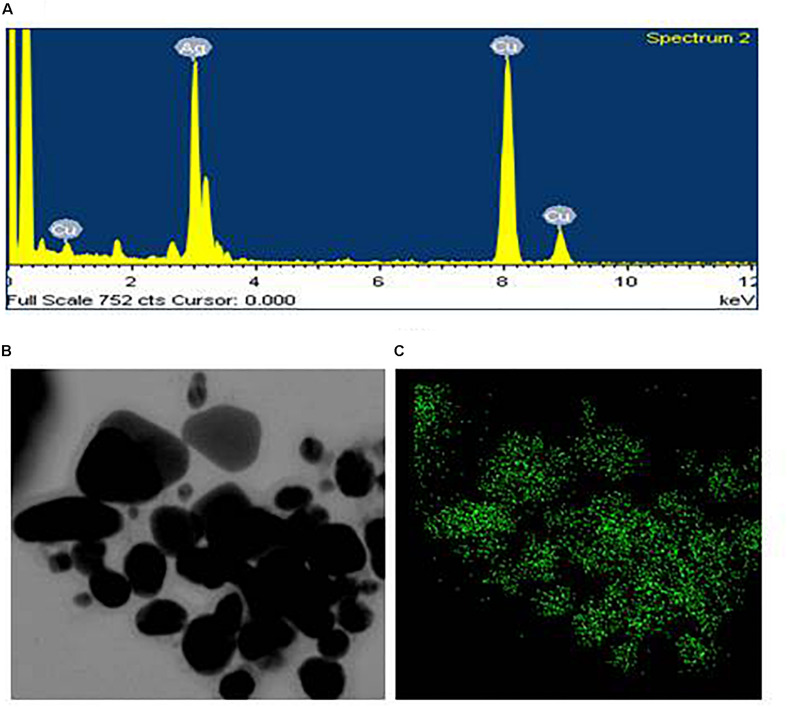
EDX spectrum of synthesized AgNPs **(A)**, FE-TEM image used for elemental mapping **(B)**, and distribution of silver in elemental mapping **(C)**.

**FIGURE 4:**
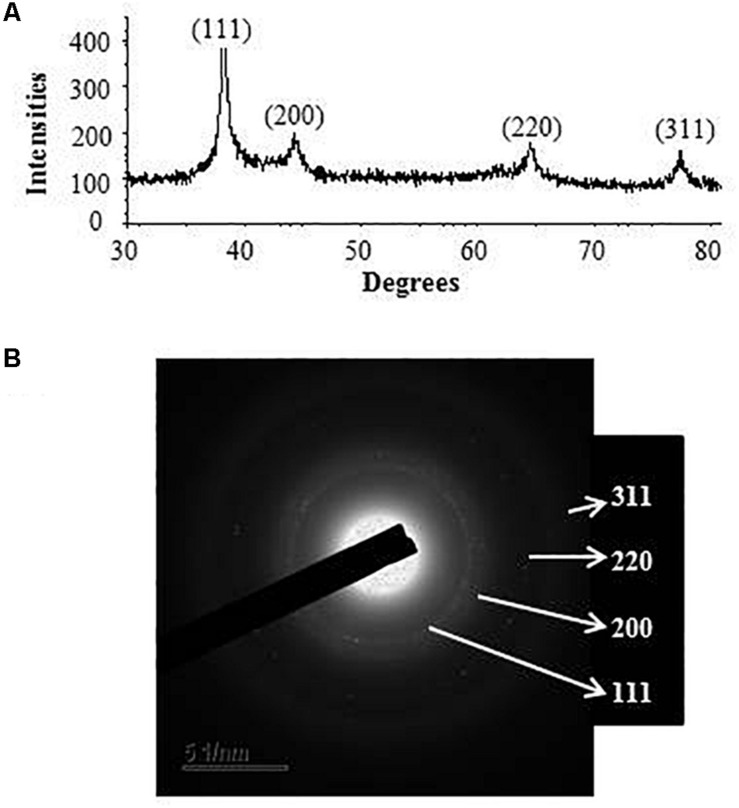
X-ray diffraction pattern **(A)** and SAED **(B)** pattern of synthesized AgNPs.

Transmission electron microscopy analysis revealed that AgNPs were spherical in shape and disperses well without notable agglomeration. The size of synthesized AgNPs was 8–30 nm (Figures 2D,E). Green AgNPs were previously synthesized using *Sphingobium* sp. (Akter and Huq, 2020), *Pseudomonas* sp. (Singh et al., 2018), and *Nostoc* sp. (Sonker et al., 2017) with sizes ranging from 7–22, 10–40, and 51–100 nm, respectively. The elemental mapping result revealed that the highest distributed element in the biosynthesized nanoproduct was silver (Figures 3A–C and Table 1). The EDX spectrum exhibited the biggest peak at 3 keV for AgNPs (Figure 3A). Some other peaks for copper and carbon were also found in the EDX pattern because of the copper grid used in FE-TEM (Figure 3A). XRD analysis revealed the peaks at two delta values of 38.12°, 44.26°, 64.41°, and 77.48°corresponding to the (111), (200), (220), and (311) reflection facets of the cubic crystalline structure of standard silver, indicated the formation of AgNPs (Figure 4A). This XRD result is similar to the result of the SAED (selected area diffraction) pattern. The crystalline nature of biosynthesized AgNPs was confirmed by the presence of a spherical ring in the SAED pattern (Figure 4B). Similar results were found in previously reported works, wherein green synthesis of AgNPs using plants and microbes has been reported (Singh et al., 2017; Huq, 2020).

**TABLE 1:** The percentage of chemical elements found in EDX spectrum of AgNPs synthesized by *Lysinibacillus xylanilyticus* MAHUQ-40.

Element	Weight%	Atomic%
Cu K	42.26	55.41
Ag L	57.74	44.59
Totals	100.00	100.00

**FIGURE 5:**
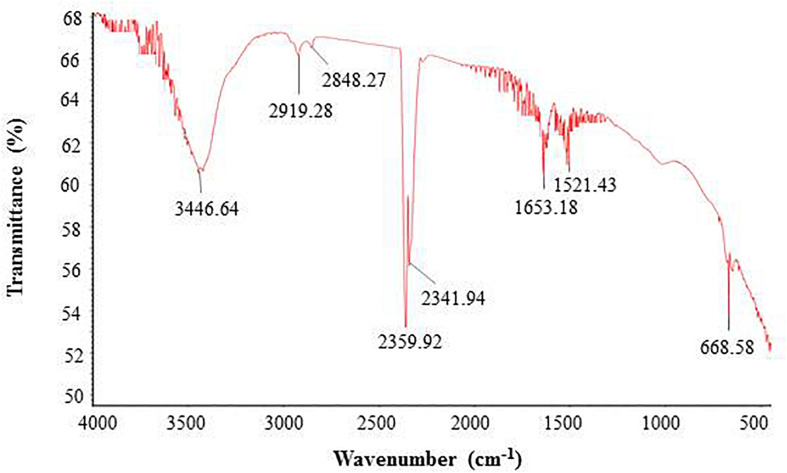
FT-IR spectrum of synthesized AgNPs.

**FIGURE 6:**
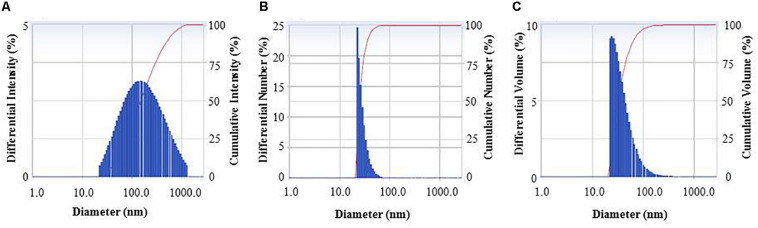
Particle size distribution of biogenic AgNPs on the basis of intensity **(A)**, number **(B)**, and volume **(C)**.

FTIR analysis revealed that different functional groups surrounded the green synthesized AgNPs. The FTIR pattern of AgNPs had several peaks at 3,446.64, 2,919.28, 2,848.27, 2,359.92, 2,341.94, 1,653.18, 1,521.43, and 668.58 cm^–1^ (Figure 5). A high absorption peak at 3,446.64 cm^–1^ corresponded to N-H (protein) stretching and O-H (polysaccharide) stretching. The absorption peaks at 2,919.28 and 2,848.27 cm^–1^ corresponded to C–H (alkane) stretching. The peaks at 2,359.92 and 2,341.94 cm^–1^ were assigned to CΞC stretching modes of vibration in alkyne groups. The bands found at 1,653.18 and 1,521.43 cm^–1^ corresponded to bending N-H stretching vibration of amine groups. In the FTIR spectrum, the presence of functional groups for proteins and polysaccharides and other functional groups indicated that these biomolecules could possibly be involved for the synthesis of AgNPs and also for the stabilization of AgNPs by creating a capping layer around the synthesized AgNPs. These findings are in accordance with the previously published works (El-Naggar et al., 2016; Hamouda et al., 2019). The DLS analysis on the basis of intensity, number, and volume revealed the AgNPs with an average diameter of 117.9 nm (Figure 6). The polydispersity index was found to be 0.332. This result is in accordance with the previously published work when synthesized using microorganisms (Singh et al., 2018).

### Antimicrobial Activity of Eco-Friendly Synthesized AgNPs

**FIGURE 7:**
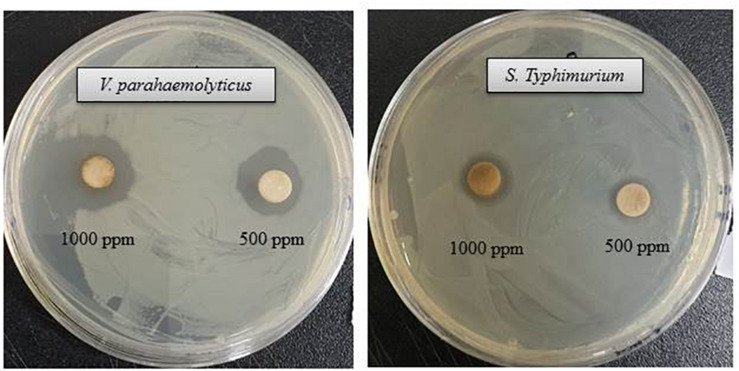
Inhibition zones of biogenic AgNPs (30 μl) at 500 and 1,000 ppm against *V. parahaemolyticus* and *S. Typhimurium.*

**FIGURE 8:**
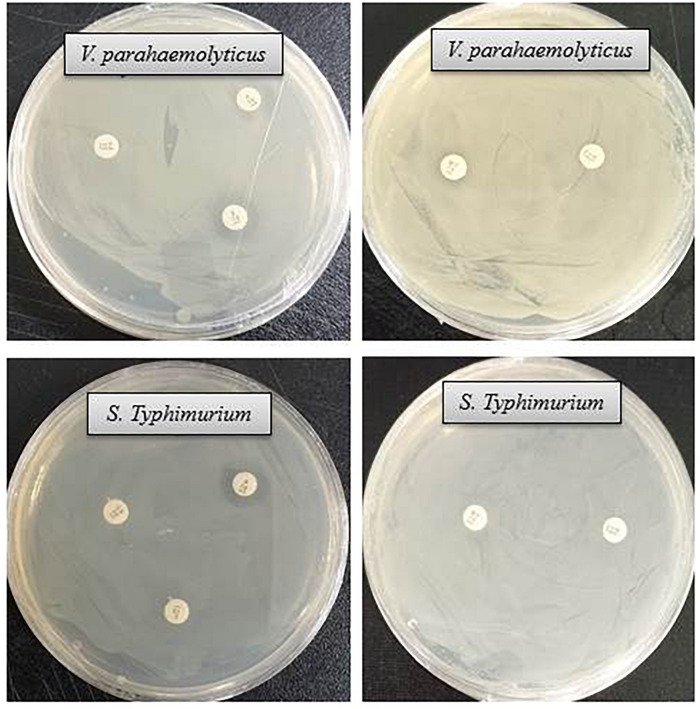
Inhibition zones of some commercial antibiotics against *V. parahaemolyticus* and *S. Typhimurium*. Abbreviation: E (erythromycin, 15 μg/disc), OL (oleandomycin, 15 μg/disc), P (penicillin, G 10 μg/disc), MY (lincomycin, 15 μg/disc), and VA (vancomycin, 30 μg/disc).

The antibacterial efficacy of the eco-friendly synthesized AgNPs was tested against two pathogenic bacteria, *V. parahaemolyticus* and *S. Typhimurium*, at both 500 ppm and 1,000 ppm concentrations (Figure 7). The green synthesized AgNPs showed significant inhibition against both pathogens (*V. parahaemolyticus* and *S. Typhimurium*) compared to the commercial antibiotics. The antibacterial activity was calculated by measuring the diameter of zone of inhibition (Table 2). Figure 7 reveals the inhibition zone around the discs supplemented with biogenic AgNPs. AgNPs showed a highest zone of inhibition of 21.4 and 14.5 mm against *V. parahaemolyticus and S. Typhimurium*, respectively. Results revealed that the eco-friendly synthesized AgNPs have strong antibacterial activity against both *V. parahaemolyticus* and *S. Typhimurium* (Figure 7). Findings of the present study suggest that the eco-friendly synthesized AgNPs were able to control the MDR pathogens *V. parahaemolyticus and S. Typhimurium*. Some commercial antibiotics including lincomycin, vancomycin, oleandomycin, erythromycin, and penicillin G were also used to evaluate their antimicrobial activity against *V. parahaemolyticus* and *S. Typhimurium*. Only, erythromycin showed very weak activity against *V. parahaemolyticus* but other four antibiotics did not show any inhibition zone. On the other hand, only penicillin G showed weak activity against *S. Typhimurium* but other four antibiotics did not show any activity against *S. Typhimurium* (Figure 8 and Table 3). The findings of the current study are in line with the results of some other studies where AgNPs were suggested as an effective antibacterial agent (Singh et al., 2018; Akter and Huq, 2020).

**TABLE 2:** Antibacterial efficacy of eco-friendly synthesized AgNPs against *V. parahaemolyticus* and *S. Typhimurium*.

Pathogenic species	Zone of inhibition (mm)	
	1,000 ppm 500 ppm	
*V. parahaemolyticus* (ATCC 17802)	21.4 ± 1.5	19.5 ± 1.3
*S. Typhimurium* (ATCC 14028)	14.5 ± 1.0	13.1 ± 1.4

**TABLE 3:** Antibacterial efficacy of commercial antibiotics against *V. parahaemolyticus* and *S. Typhimurium*.

Pathogenic species	Antibiotic	Zone of inhibition (mm)
*V. parahaemolyticus* (ATCC 17802)	Oleandomycin	-
	Erythromycin	11.5 ± 1.1
	Lincomycin	-
	Vancomycin	-
	Penicillin G	-
*S. Typhimurium* (ATCC 14028)	Oleandomycin	
	Erythromycin	-
	Lincomycin	-
	Vancomycin	-
	Penicillin G	12.2 ± 1.3

*-, no zone of inhibition.*

### Minimum Inhibitory and Minimum Bactericidal Concentration

**FIGURE 9:**
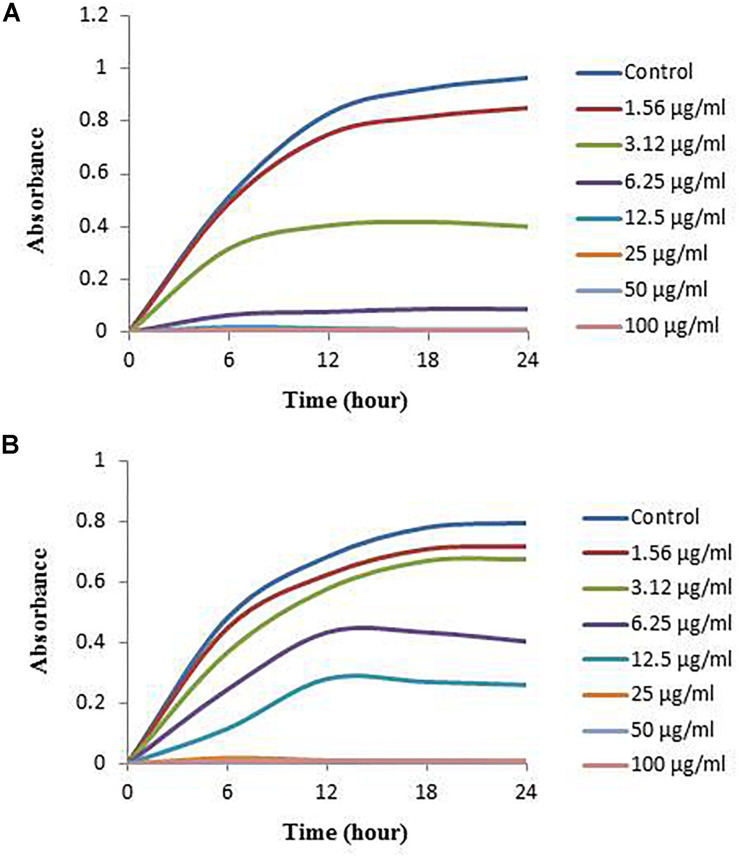
Growth curves of *V. parahaemolyticus* **(A)** and *S. Typhimurium* **(B)** cultured in NB with 3% NaCl and MHB, respectively, with different concentrations of the synthesized AgNPs to determine MIC.

**FIGURE 10:**
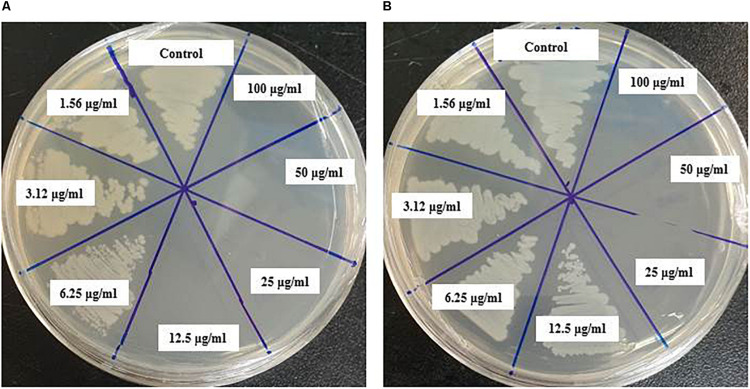
MBC of biosynthesized AgNPs against *V. parahaemolyticus* **(A)** and *S. Typhimurium* **(B)**.

In this study, the MIC and MBC of synthesized AgNPs against *V. parahaemolyticus* and *S. Typhimurium* were calculated by broth microdilution method. The eco-friendly synthesized AgNPs exhibited a MIC of 3.12 and 6.25 μg/ml for *V. parahaemolyticus* and *S. Typhimurium*, respectively. This result indicates that the biogenic AgNPs extremely suppressed the growth of both pathogens, *V. parahaemolyticus* and *S. Typhimurium* (Figures 9A,B). These MICs were significantly lower than some other antibacterial agents (Rehman et al., 2019; Guo et al., 2020; Yang et al., 2020). The MBCs of synthesized AgNPs for *V. parahaemolyticus* and *S. Typhimurium* were 12.5 and 25 μg/ml, respectively (Figures 10A,B), which were also very low compared to some other antibacterial agents (Rehman et al., 2019; Guo et al., 2020; Yang et al., 2020).

### Morphological Evaluation of Treated Cells by FE-SEM

**FIGURE 11:**
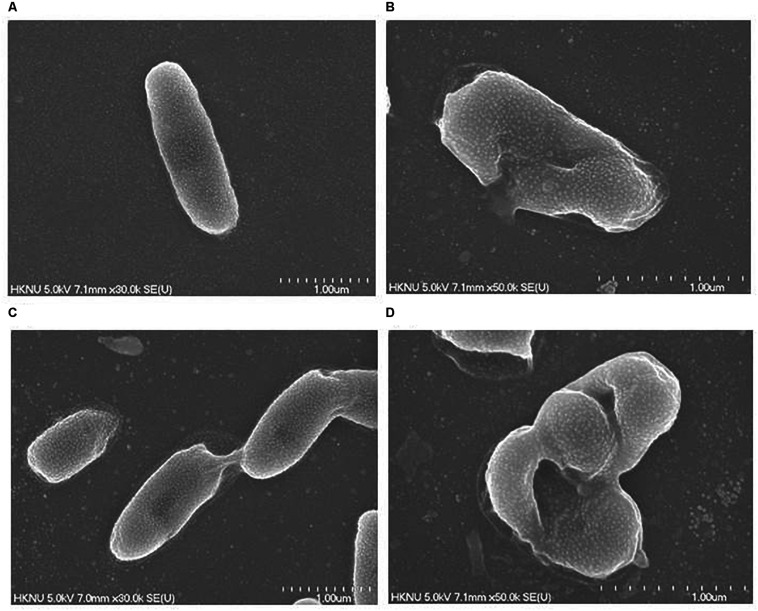
FE-SEM images of normal *V. parahaemolyticus* cells **(A)**, 1× MBC AgNP-treated *V. parahaemolyticus* cells **(B)**, normal *S. Typhimurium* cells **(C)**, 1× MBC AgNP-treated *S. Typhimurium* cells **(D)**.

The structural and morphological alterations of *V. parahaemolyticus and S. Typhimurium* cells treated with biosynthesized AgNPs were directly seen using FE-SEM (Figure 11). Through FE-SEM analysis, it was found that the untreated *V. parahaemolyticus* cells were intact, normal rod-shaped and the structural integrity of the bacterial cells was good without any damage (Figure 11A). However, after treatment with 1 × MBC of synthesized AgNPs, the shape of *V. parahaemolyticus* cells becomes abnormal, irregular, and wrinkled with the cell membrane entirely collapsed and damaged (Figure 11B). On the other hand, untreated *S. Typhimurium* cells were also found to be normal, fresh, and rod-shaped with an intact cell surface (Figure 11C). However, *S. Typhimurium* cells treated with 1 × MBC of biogenic AgNPs were shown to be abnormal, deformed, damaged, and wrinkled and with a cracked outer surface, and cell membranes were completely collapsed (Figure 11D). The treated cells displayed a serious deformation and cytoplasmic leakage, resulting in the damage of membrane integrity and the death of cells. The structural and morphological alterations, damage of bacterial cell wall, and cell membrane indicated that the eco-friendly synthesized AgNPs might interfere with the metabolic process and the normal cell functions, which led to the death of bacterial cells. Similar findings were reported in previous studies, where they explain the antibacterial mechanism of NPs against pathogenic microorganisms (Kim et al., 2007; Shankar and Rhim, 2015).

## Conclusion

Bacteria produce valuable primary and secondary metabolites including pharmaceuticals, pigments, and proteins. These bio-compounds may play an important role in AgNPs production. In the present study, we isolated and identified the strain *L. xylanilyticus* MAHUQ-40 and used their culture supernatant for eco-friendly synthesis of AgNPs. The biosynthesized AgNPs were analyzed using UV-Vis, FE-TEM, XRD, FT-IR, and DLS. FE-TEM images indicated that the AgNPs were circular and the size was 8–30 nm. The appearance of hydroxyl and amide groups in the FTIR spectrum revealed that polysaccharides and proteins were crucial factors during eco-friendly and facile synthesis of AgNPs. The extracellular method was used for the eco-friendly synthesis of AgNPs. The strategy of the present study was eco-friendly, facile, green, and rapid without the addition of any toxic dispersing or reducing agents. Moreover, the eco-friendly synthesized AgNPs showed potent antibacterial efficacy against antibiotic-resistant pathogenic strains *V. parahaemolyticus* and *S. Typhimurium*. The MICs and MBCs of the AgNPs synthesized by strain MAHUQ-40 were 3.12 and 12.5 μg/ml, respectively, against *V. parahaemolyticus* and 6.25 and 25 μg/ml, respectively, against *S. Typhimurium*. FE-TEM analysis showed that the biogenic AgNPs can cause morphological and structural alterations and destroy the membrane integrity in both pathogenic strains *V. parahaemolyticus* and *S. Typhimurium*. Here, we showed the first report for eco-friendly and facile synthesis of biogenic AgNPs using *L. xylanilyticus* MAHUQ-40. AgNPs fabricated using *L. xylanilyticus* MAHUQ-40 could be a potential antimicrobial agent in both pharmaceutical and medical industries to control the antibiotic-resistant microorganisms.

## Data Availability Statement

The datasets presented in this study can be found in online repositories. The names of the repository/repositories and accession number(s) can be found in the article/supplementary material.

## Author Contributions

MAH conceived the original screening and research plans, performed all of the experiments, and wrote the article.

## Conflict of Interest

The author declares that the research was conducted in the absence of any commercial or financial relationships that could be construed as a potential conflict of interest.
